# Predator‐driven elemental cycling: the impact of predation and risk effects on ecosystem stoichiometry

**DOI:** 10.1002/ece3.1760

**Published:** 2015-10-15

**Authors:** Shawn J. Leroux, Oswald J. Schmitz

**Affiliations:** ^1^ Department of BiologyMemorial University of NewfoundlandSt. John'sNewfoundlandA1B 3X9Canada; ^2^ School of Forestry and Environmental StudiesYale UniversityNew HavenConnecticut06511USA

**Keywords:** Carbon cycling, nitrogen cycling, physiological plasticity, predator consumptive effects, predator nonconsumptive effects, trophic cascade

## Abstract

Empirical evidence is beginning to show that predators can be important drivers of elemental cycling within ecosystems by propagating indirect effects that determine the distribution of elements among trophic levels as well as determine the chemical content of organic matter that becomes decomposed by microbes. These indirect effects can be propagated by predator consumptive effects on prey, nonconsumptive (risk) effects, or a combination of both. Currently, there is insufficient theory to predict how such predator effects should propagate throughout ecosystems. We present here a theoretical framework for exploring predator effects on ecosystem elemental cycling to encourage further empirical quantification. We use a classic ecosystem trophic compartment model as a basis for our analyses but infuse principles from ecological stoichiometry into the analyses of elemental cycling. Using a combined analytical‐numerical approach, we compare how predators affect cycling through consumptive effects in which they control the flux of nutrients up trophic chains; through risk effects in which they change the homeostatic elemental balance of herbivore prey which accordingly changes the element ratio herbivores select from plants; and through a combination of both effects. Our analysis reveals that predators can have quantitatively important effects on elemental cycling, relative to a model formalism that excludes predator effects. Furthermore, the feedbacks due to predator nonconsumptive effects often have the quantitatively strongest impact on whole ecosystem elemental stocks, production and efficiency rates, and recycling fluxes by changing the stoichiometric balance of all trophic levels. Our modeling framework predictably shows how bottom‐up control by microbes and top‐down control by predators on ecosystems become interdependent when top predator effects permeate ecosystems.

## Introduction

Trophic transfer and recycling of elements are integral parts of a fundamental ecosystem process that determines rates of primary and secondary production, food chain length, trophic biomass, and species diversity (DeAngelis 1992; Bardgett and Wardle 2010; Loreau 2010). Consumers can mediate elemental transfer and recycling through resource consumption as well as through the release of elements as byproducts of their physiology (Kitchell et al. 1979; DeAngelis 1992; Vanni 2002; Schmitz et al. 2010; Dalton and Flecker 2014). Ecological stoichiometry has enhanced understanding of the mechanisms driving consumer‐mediated elemental transfer and recycling by explicitly connecting organismal‐based physiology to this whole ecosystem process (Sterner and Elser 2002).

Current stoichiometric theory largely holds that in plant‐based food chains of terrestrial ecosystems, the rate of elemental transfer up the food chain is primarily constrained by a mismatch between herbivore nutritional demands and the nutritional quality of their plant resources. Herbivores have high demands for dietary N to support growth and reproduction and must regulate body elemental contents within low C:N levels (Elser et al. 2000; Fagan et al. 2002; Raubenheimer et al. 2009). Yet, they must select their diets from plant resources that tend to have high C contents (dominated by indigestible C‐based compounds) and comparatively low N (Robbins 1983; Karasov and Martinez del Rio 2007). This mismatch creates a bottleneck in the of rate elemental transfer up the food chain. The transfer of elements further up the chain to predators is held to be less constrained because herbivore and predator elemental demands are more closely matched. In such a conception, any top‐down ecosystem level feedbacks come about through recycling of elements that are released directly from herbivores and predators back to the soil nutrient pool (Kitchell et al. 1979; DeAngelis 1992; Vanni 2002; Schmitz et al. 2010).

However, herbivores, by virtue of occupying intermediate trophic levels within food chains, must cope with the dual pressures of selecting plant resources while avoiding becoming resources for predators (Kitchell et al. 1979; Pomeroy 2001; Schmitz et al. 2008, 2010). Evading predation can reduce foraging effort, which may also constrain the transfer rate of elements up the trophic chain (Trussell et al. 2006). The perceived risk of predation can also induce chronic physiological stress responses that elevate herbivore metabolic rate (Hawlena and Schmitz 2010a; Zanette et al. 2011; Thaler et al. 2012; Clinchy et al. 2013). This keeps herbivores in a heightened state of alertness to increase the chance they can escape predators under chronic risk (Hawlena and Schmitz 2010b; Zanette et al. 2011; Clinchy et al. 2013). But, elevated metabolism (respiration) can increase nutrient demand for energy containing soluble carbohydrate C (McPeek et al. 2001; Hawlena and Schmitz 2010a), which also tends to be limiting in terrestrial ecosystems (Robbins 1983; Karasov and Martinez del Rio 2007). Such heightened respiration can result in declining secondary production (Trussell et al. 2006; Trussell and Schmitz 2012). Hence predation risk may create another kind of bottleneck in trophic transfer. Furthermore, a diet shift in favor of C may cause dietary N intake to be in excess, because the amount of C available for production correlates positively with N (Sterner and Elser 2002). Stressed herbivores should then release N to avoid incurring toxicity effects (Sterner and Elser 2002; Hawlena and Schmitz 2010b). Thus, physiological responses of herbivores to perceived predation risk could trigger additional top‐down feedback that alters the amount and balance of C and N entering the soil pool in inorganic (excreted N) and organic (plant and animal detritus) form (Hawlena and Schmitz 2010a; Leroux et al. 2012), with attendant significant affects on organic matter decomposition rate (Hawlena et al. 2012).

Analyses of such predator effects on elemental transfer and recycling have tended to consider predation (consumptive) effects independently of predation risk effects (e.g., DeAngelis 1992; Hall et al. 2007; Hall 2009; Schmitz et al. 2010; Bassar et al. 2012; Leroux et al. 2012). Yet both must operate simultaneously otherwise predators that merely cause risk effects would starve to death. The challenge, however, is to understand the interplay between these two effects and quantify their relative impact on ecosystem processes (Bolker et al. 2003; Schmitz 2010). The predictive theory needed to motivate empirical analyses is, however currently lacking. To this end, we elaborate and analyze a series of models to explore how predation and risk effects independently and in combination determine the capacity for predators to control elemental cycling.

## The Theoretical Framework

Our models are designed to help organize thinking about how predators may influence nutrient cycling. They are based on fundamental principles of elemental flux and storage among different trophic levels in an ecosystem, based on known mechanisms for their action (Leroux and Loreau 2010; Schmitz et al. 2010). But, they intentionally do not contain mechanistic detail needed to depict any one specific, real system because such details are lacking for most systems (Schmitz et al. 2014). Instead, the models embody many of the qualitative mechanisms that apply broadly across terrestrial ecosystems. By doing this, we hope to inspire quantitative empirical measurements of predator effects in all kinds of ecosystem types.

**Figure 1:**
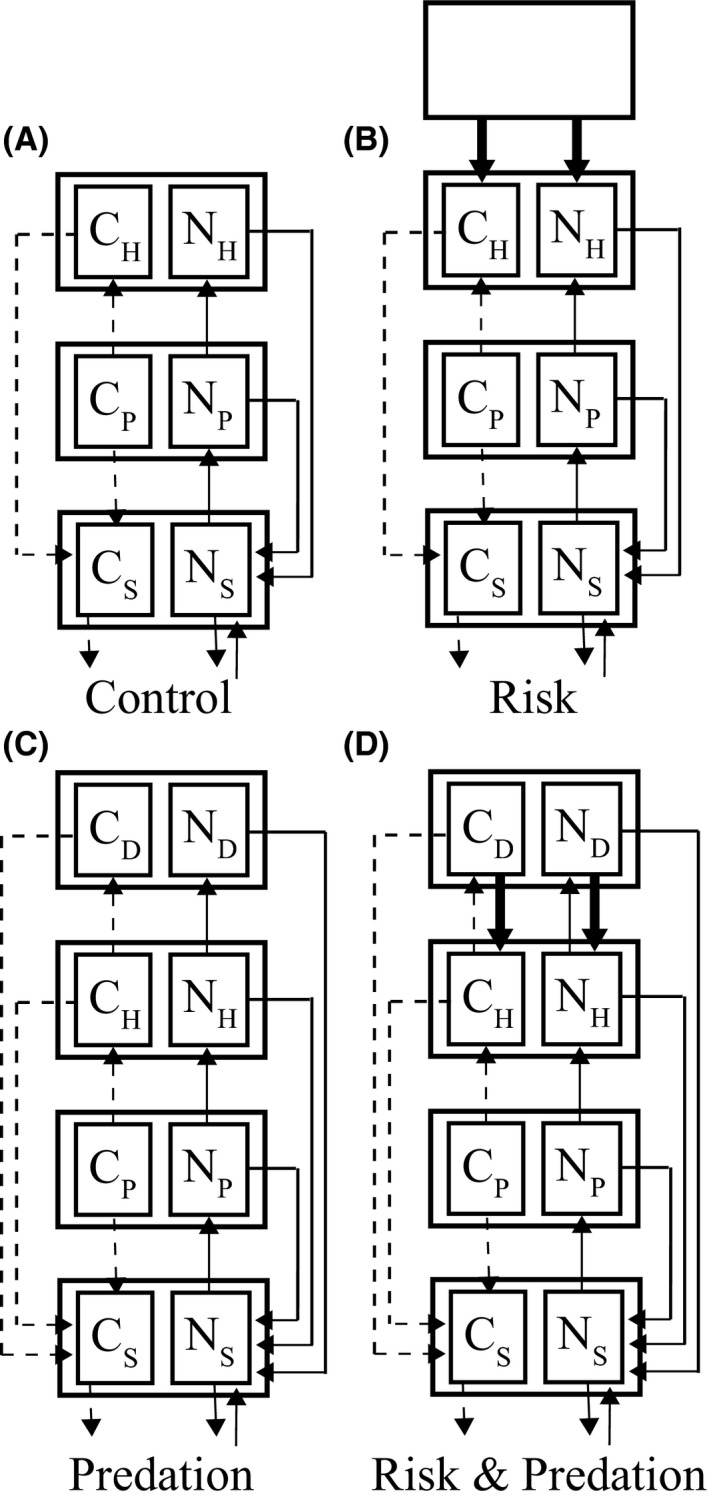
Conceptual diagram of our four general ecosystem models; (A) “Control” a soil‐plant‐herbivore model without a predator trophic level, (B) “Risk” a soil‐plant‐herbivore model with predation risk, (C) “Predation” a soil‐plant‐herbivore‐predator model with predators that do not impose risk, and (D) “Risk & Predation” a soil‐plant‐herbivore‐predator model with predators that have consumptive and risk effects. The models track the quantity of *C*_*i*_ and *N*_*i*_ among soil (*i* = *S*), plant (*i* = *P*), herbivore (*i* = *H*), and predator (*i* = *D*) compartments. Thick downward arrows represent predation risk. See Table 1 for variable and parameter definitions and Figure A1 for a detailed diagram of our model.

At their core, the models embody the conventional ecosystem compartment structure that includes soil elemental pools, plants, herbivores, and predators (Fig. 1) often used when examining organismal effects on ecosystem functioning (Hall et al. 2007; Leroux and Loreau 2010; Loreau 2010; Bassar et al. 2012; Leroux et al. 2012). The models capture the essential features of elemental cycling (DeAngelis 1992; Moore et al. 2004; Loreau 2010), including elemental uptake by plants from the abiotic environment (i.e., carbon uptake from the atmosphere and nitrogen uptake from soils) and elemental transfer and loss to and from all compartments through trophic interactions, respiration, excretion, egestion, and leaching out of the ecosystem. As such, the models depict open systems, i.e., elements are not solely recycled within the confines of the ecosystem. Nevertheless, they are formulated to obey fundamental mass balance requirements (Loreau 2010) such that, at equilibrium, elemental inputs to the ecosystem equal elemental losses from the ecosystem plus storage.

We use a stoichiometric approach that focuses on fluxes and pool sizes of nitrogen (N) and carbon (C), because these appear to be among the most important elements in terrestrial ecosystems (Elser et al. 2000; Fagan et al. 2002). Nevertheless, the principles explored in these models could easily be extended to considerations of other important elements such as phosphorus.

In general, predators can determine the fate of C and N within ecosystems by causing changes in elemental distribution among different trophic compartments or by acting as vectors that translocate elements spatially between ecosystems (Vanni 2002; Hall et al. 2007; Leroux and Loreau 2010; Schmitz et al. 2010). Our focus here is on how predators affect the distribution of elements among trophic compartments within ecosystems. We therefore assume that predators do not translocate nutrients by migrating into or out of the ecosystems.

Within our model ecosystems, predators instigate their effects in two ways (Abrams 2007). They kill and consume prey, and the strength of this interaction determines the flux rate of elements from herbivores to predators, and hence the amount of elements stored in the predator trophic level and released from it via respiration, excretion, and egestion. Predators can also have nonconsumptive risk effects that changes the rate of plant consumption by herbivores, and hence the flux rate of elements into the herbivore trophic level. Perceived predation risk also induces chronic stress in herbivores, which changes herbivore elemental demand to support higher maintenance costs at the expense of production. This influences herbivore elemental uptake from plants, and elemental release via respiration, excretion, and egestion, which in turn alters the balance of elements taken up by predators.

The mechanisms of predator effect are explored by building upon an earlier model that just examined the implications of heightened herbivore metabolism due to predation risk on N and C cycling (Leroux et al. 2012). Our current approach represents a significant advance from this earlier formalism in two respects. First, it explicitly considers elemental flows through a functional predator trophic level in which predators can have consumptive and nonconsumptive effects. Second, we implement an altogether different mechanism for nutrient allocation between competing demands of maintenance and production. Muller et al. (2001) identify two options for implementing maintenance costs in ecological models: (1) debit the expenditure from that assimilated to meet maintenance before biomass is formed or (2) add a loss term accounting for respiration of biomass for maintenance purposes. Most models apply method (2) by accounting for maintenance costs via respiration and recycling (e.g., Daufresne and Loreau 2001; Loreau 2010). But, this approach cannot deal with the trade‐off between the competing demands of maintenance and production that is faced when herbivores become stressed. We thus consider the physiological trade‐off by using a convention from dynamic energy budget models that instantaneously debit from assimilation to meet elemental demands for maintenance (including stress responses and activity, collectively referred to here as active metabolism) prior to biomass formation, and production (Kuijper et al. 2004; Hall 2009). Accordingly, we assume that herbivores and predators take up a certain quantity of N and C per unit time from plants and herbivores. A portion of the herbivore (or, predator) assimilated N and C, *ρ* (or, *ε*)*,* is used for active metabolism, and the rest, 1 − *ρ* (or, 1 − *ε*), goes toward growth and reproduction. As more resources are devoted to active metabolism, less is available for growth and reproduction.

### The model

Consistent with previous analyses of elemental cycling (Moore et al. 2004; Leroux and Loreau 2010; Loreau 2010) we take a minimalist approach to describe system dynamics. Our model structure embodies the least number of equations needed to explicitly track C and N fluxes and storage among the four focal trophic compartments (Fig. 1) while representing the salient parts of the trophic transfer and recycling process. Thus we deploy the following core set of differential equations to account for the stock size and flux of *C*_*i*_ and *N*_*i*_ among soil (*i = S*), plant (*i = P*), herbivore (*i* = *H*), and predator (*i* = *D*) compartments within the ecosystem, and losses from the ecosystem due to respiration of C and leaching of C and N. (Table 1 summarizes the variable and parameter definitions and their units):

(1a) $$\frac{dN_{S}}{dt}=1-kN_{S}+r_{P}N_{P}+r_{H}N_{H}+r_{D}N_{D}+\rho a_{H}N_{P}N_{H}+\varepsilon a_{D}N_{H}N_{D}+W_{\mathrm{NH}}-a_{P}C_{S}N_{S}N_{P}$$

(1b) $$\frac{dC_{S}}{dt}=\psi \alpha r_{P}N_{P}+\beta r_{H}N_{H}+\beta r_{D}N_{D}+\left(1-\mu \right)\rho \psi \alpha a_{H}N_{P}N_{H}+\left(1-\tau \right)\varepsilon \beta a_{D}N_{H}N_{D}-qC_{S}$$

(2a) $$\frac{dN_{P}}{dt}=a_{P}C_{S}N_{S}N_{P}-r_{P}N_{P}-a_{H}N_{P}N_{H}$$

(2b) $$\frac{dC_{P}}{dt}=\psi \alpha a_{P}C_{S}N_{S}N_{P}-\psi \alpha r_{P}N_{P}-\psi \alpha a_{H}N_{P}N_{H}$$

(3a) $$\frac{dN_{H}}{dt}=\left(1-\rho \right)a_{H}N_{P}N_{H}-r_{H}N_{H}-W_{\mathrm{NH}}-a_{D}N_{H}N_{D}$$

(3b) $$\frac{dC_{H}}{dt}=\psi \alpha \left(1-\rho \right)a_{H}N_{P}N_{H}-\beta r_{H}N_{H}-W_{\mathrm{CH}}-\beta a_{D}N_{H}N_{D}$$

(4a) $$\frac{dN_{D}}{dt}=\left(1-\varepsilon \right)a_{D}N_{H}N_{D}-r_{D}N_{D}$$

(4b) $$\frac{dC_{D}}{dt}=\beta \left(1-\varepsilon \right)a_{D}N_{H}N_{D}-\beta r_{D}N_{D}$$

**Table 1:** State variable, parameter, and function definitions and dimensions for our stoichiometrically explicit model

Symbols	Definitions	Dimension
Variables		
*N*_S_	Nitrogen stock in soils	Quantity of nutrient
*C*_S_	Carbon stock in soils	Quantity of nutrient
*N*_P_	Nitrogen stock in plants	Quantity of nutrient
*C*_P_	Carbon stock in plants	Quantity of nutrient
*N*_H_	Nitrogen stock in herbivores	Quantity of nutrient
*C*_H_	Carbon stock in herbivores	Quantity of nutrient
*N*_D_	Nitrogen stock in predators	Quantity of nutrient
*C*_D_	Carbon stock in predators	Quantity of nutrient
Parameters		
*I*	Constant nitrogen input rate to soils	Time^−1^·quantity of nutrient
*k*	Nitrogen loss rate from soils	Time^−1^
*q*	Carbon loss rate from soils	Time^−1^
*a*_P_	Nitrogen mineralization rate	Time^−1^·quantity of nutrient^−2^
*a*_H_	Herbivore uptake rate	Time^−1^·quantity of nutrient^−1^
*a*_D_	Predator uptake rate	Time^−1^·quantity of nutrient^−1^
*r*_P_	Nitrogen recycling rate of plants	Time^−1^
*r*_H_	Nitrogen recycling rate of herbivores	Time^−1^
*r*_D_	Nitrogen recycling rate of predators	Time^−1^
*ρ*	Proportion of Nitrogen consumed by herbivores that is used for active metabolism. (1* − ρ*) is used for growth and reproduction	Dimensionless; 0 < *ρ *< 1
*ε*	Proportion of Nitrogen consumed by predators that is used for active metabolism. (1* − ε*) is used for growth and reproduction	Dimensionless; 0 < *ε *< 1
*μ*	Proportion of Carbon respired by herbivores	Dimensionless; 0 < *μ *< 1
*τ*	Proportion of Carbon respired by predators	Dimensionless; 0 < *τ *< 1
*ψ*	Proportion of Carbon that is soluble	Dimensionless; 0 < *ψ *< 1
*α*	*C*_P_:*N*_P_ ratio	Dimensionless
*β*	*C*_H_:*N*_H_ ratio and *C*_D_:*N*_D_ ratio	Dimensionless
Functions		
*W*_NH_	Herbivore differential assimilation rate of Nitrogen. W_NH_ * = *(1* − ρ*)*a*_H_ *N*_P_ *N*_H_((*β − ψα*)/*β*)	
*W*_CH_	Herbivore differential assimilation rate of Carbon. *W*_CH_ * = *(1* − ρ*)*a*_H_ *N*_P_ *N*_H_(*ψα − β*)	

The equations couple C and N cycles because of the stoichiometric requirement that both elements are needed to balance demands for maintenance and production. Moreover, herbivores and predators consume both elements together in biochemicals. One could track them together as a ratio (e.g. Loladze et al. 2000). However, we tracked them separately because they can be differentially assimilated and released as metabolic rates change with herbivore stress from predation risk. We explicitly track and quantify the fate of the soluble fraction of C (*ψC*) through the trophic compartments; the fate of the recalcitrant fraction of C is implicitly quantified as (1 − *ψ*)*C*. We can modify the fraction of C that is soluble in our ecosystems by varying the magnitude of *ψ*. Doing this allows us to examine the implications of resource nutritional quality on trophic control of ecosystems (c.f. Hall et al. 2007). But it further allows us to examine the effects of interactions between nutritional quality and changing herbivore metabolic demand for C and N in response to predation risk.

We assume that physical and biotic processes determine the pool size of soil N. Physical processes include external input (*I*) and loss due to soil leaching (*kN*_S_) (Chapin et al. 2011). Biotic inputs come from recycling (DeAngelis 1992). We specify a baseline N input from dead plant, herbivore and predator matter (*r*_P_ *N*_P_, *r*_H_ *N*_H_, *r*_D_ *N*_D_). We allow further inputs to soil N from herbivores due to changing assimilation rates from altered metabolic demand for N and C (*W*_NH_ *= *(*1 − ρ*)*a*_H_ *N*_P_ *N*_H_((*β − ψα*)/*β*); where adjustments to baseline soil N input depends on the proportional difference (*β − ψα*)/*β* between the C:N ratio needed to meet herbivore elemental demand *β* and the fraction of soluble C (*ψα*; and hence potential excess N) obtained from plants given an ambient plant C:N ratio *α*. Additional inputs to soil N come from metabolic waste released by herbivores and predators (ρ*a*_H_ *N*_P_ *N*_H_, ε*a*_D_ *N*_H_ *N*_D_), where ρ*a*_H_ and ε*a*_D_ are respectively the proportion of herbivore and predator N uptake per unit time that is used for active metabolism which conforms to the assumption that elemental demands for maintenance are instantaneously debited from assimilated nutrients (Kuijper et al. 2004; Hall 2009). Finally, soil N is lost due to plant uptake following N mineralization (*a*_P_ *C*_S_ *N*_S_ *N*_P_) where *a*_P_ is the soil mineralization rate. Consistent with empirical evidence (Reinertsen et al. 1984; Gilmour et al. 1985; Ekblad and Nordgren 2002; Weintraub and Schimel 2003; Buchkowski et al. 2015), we assume mineralization rate is dependent on soil C as well as N.

We assume that the soil C stock is determined by baseline inputs of dead plant, herbivore and predator matter (*αr*_P_ *N*_P_, *βr*_H_ *N*_H_, *βr*_D_ *N*_D_; Facelli and Pickett 1991; Chapin et al. 2011), and by inputs from nonrespired herbivore and predator metabolic wastes ((*1 − μ*)*ρψαa*_H_ *N*_P_ *N*_H_ + (*1 − τ*)*εβa*_D_ *N*_H_ *N*_D_; Zanotto et al. 1997). Finally, soil C is lost from the ecosystem by leaching (*qC*_S_; Chapin et al. 2011).

We assume that plants take up mineralized inorganic N from soil pools (*a*_P_ *C*_S_ *N*_S_ *N*_P_) with plant N losses due to background mortality (*r*_P_ *N*_P_) and herbivory (*a*_H_ *N*_P_ *N*_H_). We assume plants take up atmospheric C (i.e., CO_2_) for photosynthesis and combine soil N uptake to create plant biomass with a C:N ratio *α* of which the fraction *ψ* is soluble C. Hence, we allow for stoichiometric plasticity of plants by letting the proportion of plant C that is soluble (i.e., *ψ*) vary. Plant biomass C also is taken up by herbivory (ψ*αa*_H_ *N*_P_ *N*_H_).

As described above, a portion (1 − *ρ*) of the herbivore assimilated N (*a*_H_ *N*_P_ *N*_H_) and soluble C (*ψαa*_H_ *N*_P_ *N*_H_) is taken up and combined to form herbivore biomass (see below for further details on uptake rates). Herbivores are assumed to recycle N and C through baseline egestion, excretion and natural mortality (Vanni 2002; Bump et al. 2009; Schmitz et al. 2010). The quantity of C or N egested and excreted can vary because of differential assimilation to maintain homeostasis. Herbivores also recycle C at a constant C:N ratio. A portion of the soluble C is respired by herbivores (*μ*); we assume the remainder (1* − μ*) is recycled to the soil carbon pool. We assume that herbivores also respire C, if in excess, to maintain homeostasis (Zanotto et al. 1997).

Similar to herbivores, a portion (1 − *ε*) of the predator assimilated N (*a*_D_ *N*_H_ *N*_D_) and soluble C (*βa*_D_ *N*_H_ *N*_D_) is taken up and combined to form predator biomass (see below for further details on uptake rates). N and C are recycled to the soil at rates *r*_D_ *N*_D_ and β*r*_D_ *N*_D_ respectively. A portion of the soluble C is respired by predators (*τ*); we assume the remainder (1 − *τ*) is recycled to the soil carbon pool.

### Plant, herbivore, and predator C:N regulation

We assume that plants, herbivores and predators maintain a homeostatic balance of C:N. But, the exact balance will change between risk and risk free conditions. Plant homeostasis implies d*C*_P_/d*t = ψα*(d*N*_P_/d*t*). Herbivore homeostasis implies d*C*_H_/d*t = β*(d*N*_H_/d*t*) where *β* differs as metabolic demand changes between risk free and risk conditions. Predator homeostasis implies d*C*_D_/d*t = β*(d*N*_D_/d*t*). We assume predator *β* is on the same order as herbivores based on similarity of animal body composition (Elser et al. 2000).

Herbivore stoichiometric plasticity in response to elevated metabolism is modeled through differential assimilation of nutrients. Under predation risk, we assume, based on empirical evidence (Hawlena and Schmitz 2010a; Zanette et al. 2011; Thaler et al. 2012; Clinchy et al. 2013), that changes in herbivore metabolic rate due to predation stress does not rise monotonically, but rather jumps discontinuously to a higher level (e.g., 45% difference between stress and stress‐free conditions [Hawlena and Schmitz 2010a]). This causes a jump in demand for soluble *C*_H_ to fuel the increased metabolism and causes excess *N*_H_ to be excreted. Consequently, under predation risk, *W*_CH_ *= 0*. Substituting *W*_CH_ *= 0* into equation d*C*_H_/d*t* provides the flux of *N*_H_ excreted by herbivores under predation risk to maintain their demand for C:N, *β*:* W*_NH_ *= *(1 − ρ)*a*_H_ *N*_P_ *N*_H_(β − ψ*α*/β). Consequently, *β > ψα*. When there is no predation risk, we assume that herbivores are limited by nitrogen to fuel their growth and maintenance and they respire the excess *C*_H_ in their diet (Zanotto et al. 1997). Consequently, with no predation risk, *W*_NH_ *= 0*. Substituting *W*_NH_ *= 0* into equations d*N*_S_/d*t* and d*N*_H_/d*t* provides the flux of *C*_H_ respired by the herbivore with no predation risk to maintain its homeostatic ratio, *β*:* W*_CH_ *= *(1 − ρ)*a*_H_ *N*_P_ *N*_H_(ψ*α* − β). Consequently *ψα > β*.

### Herbivore and predator uptake rates

We assume that herbivore elemental uptake, *a*_H_ *N*_P_ *N*_H_, can be described by a linear consumption function, where *a*_H_ is the herbivore ingestion rate of *N* and *ψαa*_H_ is the ingestion rate of soluble C. We likewise model predator uptake of N and C as *a*_D_ *N*_H_ *N*_D_, where *a*_D_ is the predator ingestion rate of *N* and *βa*_D_ is the ingestion rate of soluble C. A linear consumption function implies that there will be no upper limits to uptake, which contrasts with other models that explicitly limit consumer uptake by using saturating consumption functions (see Loladze et al. 2000; Leroux et al. 2012). But saturating functions invoke density dependence in the process of resource uptake, which cannot be invoked for elemental uptake because elements do not physically interact in this way (Loreau 2010). In our models, an upper limitation is instead imposed implicitly via the assimilation rate of soluble N and C per unit plant or animal matter ingested. This upper limit varies with the proportion (1 − *ψ*) of recalcitrant C in the diet.

## Model Analysis

We adopt the approach advanced by Bassar et al. (2012) that quantifies and compares the effects of predator‐induced changes in prey phenotypic traits on elemental pool sizes and flux within an ecosystem. In our particular case, we quantify risk and predation effects by systematically analyzing predator consumptive (Predation) and nonconsumptive (Risk) effects singly and in combination, through changes in herbivore metabolism (phenotypic plasticity). Doing this requires formulating three kinds of model scenarios. A model ecosystem with just “Predation” (soil‐plant‐herbivore‐predator model with predators that do not impose risk) quantifies the effects of direct uptake of herbivore biomass N and C (Figs. 1, A1). A model ecosystem with just “Risk” effects (soil‐plant‐herbivore model with predation risk) quantifies the effects of heightened herbivore metabolism that changes herbivore demand for N and C (Figs. 1, A1). An ecosystem with “Risk & Predation” together (soil‐plant‐herbivore‐predator model with predators that have consumptive and risk effects) quantifies effects by combining herbivore consumption and heightened herbivore metabolism (Figs. 1, A1). We further compare these three scenarios with a “Control” (soil‐plant‐herbivore model without a predator trophic level) where the predator trophic level, and hence predator effects, are absent from the ecosystem (i.e., a soil‐plant‐herbivore model without a predator trophic level).

Our goal was to understand the consequences of different kinds of predator impacts on the stocks of elements among trophic compartments under equilibrium (steady state) conditions. We did this using a hybrid analytical‐numerical approach (Hall et al. 2007; Bassar et al. 2012; McCann 2012). We began by setting the time derivatives for the systems of equations for each of the four different model scenarios to zero and identified all equilibria (presented in Appendix B). Although the models had multiple mathematically feasible equilibria, we analytically determined the feasibility conditions for the single biologically plausible equilibrium for each model scenario, viz. the equilibrium for which all trophic levels that were part of the particular model ecosystem (Figs. 1, A1) persisted (i.e., the equilibrium stock of *N*_*i*_ and *C*_*i*_ > 0).

We then quantified the fate of C and N numerically. We maintained biological realism by choosing initial parameter values for C:N ratios for our different treatments based on data from cross‐ecosystem empirical syntheses of organismal C:N ratios. Specifically, we sought to maintain two key properties of terrestrial ecosystems: (1) terrestrial plants most often have higher C:N than terrestrial invertebrate herbivores (see review in Elser et al. 2000) and (2) terrestrial invertebrate herbivores under risk have higher body C:N than conspecifics not experiencing risk (see review in Hawlena and Schmitz 2010b). Elser et al. (2000) reported a mean terrestrial plant C:N* = *36 (standard deviation, SD* = *23) and a mean terrestrial invertebrate herbivore C:N* = *6.5 (SD* = *1.9). Syntheses of C:N for risk versus no risk conditions are unavailable, but Hawlena and Schmitz (2010b) reported mean terrestrial invertebrate (i.e., grasshopper) C:N under no risk that is 0.93 × than with risk (C:N of 4.0 vs. 4.3). Based on these empirical data, we investigated ecosystem dynamics for nine different parameter sets of plant (i.e., *α*) and herbivore C:N (i.e., *β*). We present results for our “mean” parameter set where plant C:N* = *36, herbivore under risk C:N* = *6.5 and herbivores under no risk C:N* = *6.05 (0.93 × herbivores under risk). We investigate the sensitivity of our results to all combinations of mean plant C:N ± 1 SD (i.e., 23) and mean herbivore (risk and no risk) C:N ± 1 SD (i.e., 1.9). The nine combinations of parameters can be found in Appendix C Table C1.

We randomly selected from a uniform distribution all other parameter sets such that they met the feasibility conditions (i.e., equilibrium *N*_*i*_ and *C*_*i*_ > 0) for each experimental treatment. Parameters *ρ*,* ε*,* μ*,* τ*, and *ψ* are proportions constrained between 0 and 1 and all other parameters (except *α* and *β* described above) were scaled between 0 and 10. We randomly selected 1000 parameter sets from a latin‐hypercube sampling scheme with 100 equally probable bins to calculate medians and variances in ecosystem properties and functions for the different treatments. We followed recent advice from White et al. (2014) and report the magnitude of ratios in median ecosystem properties between treatments (i.e., Effect sizes, specifically Median X/Median Y). We report the magnitude of ratios in log_2_ (Biomass, Flux) and log_10_ (Production, Efficiency) between treatments but our qualitative results are robust to different log transformations. Magnitudes >1 indicate a positive effect of a treatment on an ecosystem property relative to another treatment and magnitudes <1 indicate a negative effect.

### Quantifying ecosystem properties and functions

We calculated the elemental stock sizes, production and ecological efficiency, and elemental fluxes from trophic compartments to the soil, which are considered key ecosystem functions or properties (Chapin et al. 2011). Elemental stocks were quantified as the mass of C and N at equilibrium in different trophic compartments of the ecosystems (Table D1). Production is defined as the amount of C and N allocated to plant (primary), herbivore (secondary), and predator (tertiary) biomass at equilibrium (Table D1). We investigated if any bottlenecks in elemental transfer arose by quantifying ecological efficiency as the ratio of production from one trophic level to production of the next lowest trophic level (Loreau 2010) as we move up the food chain (Table D1). Because carbon mirrors production and ecological efficiencies of nitrogen through fixed C:N ratios, we based our calculations on production and ecological efficiencies of N (Table D1). We calculated the total flux of C and N from all biotic compartments to the soil and the organism‐specific contributions using formulas presented in Table D1.

## Results

Metabolic rate was allowed to vary based on random parameter selections that fulfilled feasibility conditions with different initial treatments. We therefore validated that herbivore respiration was indeed higher in risk conditions by calculating the median and variance in herbivore metabolic rate from the random parameter selections. Figure E1 reveals that herbivore respiration is indeed higher (3‐18X) in treatments with risk (i.e., “Risk” and “Risk & Predation”) than without risk (i.e., “Control” and “Predation”).

### Risk effects on ecosystem properties and functions

**Figure 2:**
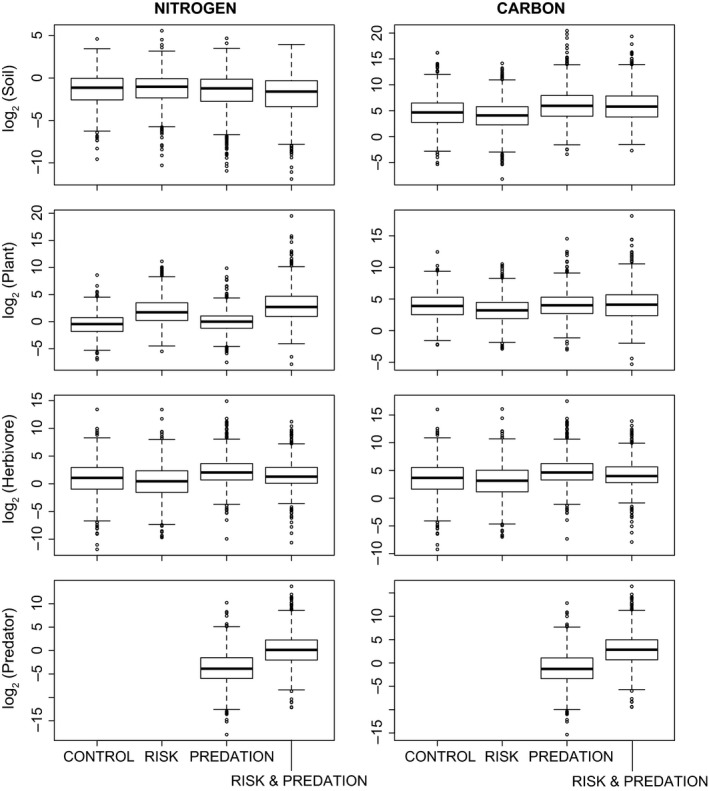
log_2_ of the mass of soil, plants, herbivores, and predators for four different models (“Control”= 3‐level no risk, “Risk”* = *3‐level with risk, “Predation”* = *4‐level no risk, “Risk & Predation”* = *4‐level with risk). Panels on the left are for nitrogen and panels on the right are for carbon. Results are for 1000 random parameter (uniform distribution) sets that meet feasibility conditions (i.e., equilibrium stocks of *N*_*i*_ and *C*_*i*_ > 0) and risk models: *α* = 36, *β* = 6.5, no risk models: *α* = 36, *β* = 6.05.

**Figure 3:**
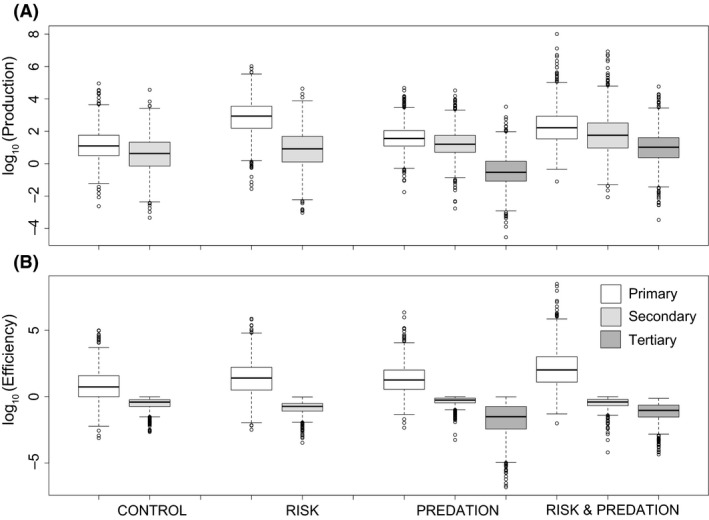
(A) log_10_ of primary, secondary, and tertiary production and (B) primary, secondary, and tertiary ecological efficiency for four different models (“Control”* = *3‐level no risk, “Risk”* = *3‐level with risk, “Predation”* = *4‐level no risk, “Risk & Predation”* = *4‐level with risk). Results are for 1000 random parameter (uniform distribution) sets that meet feasibility conditions (i.e., equilibrium stocks of *N*_*i*_ and *C*_*i*_ > 0) and risk models: *α* = 36, *β* = 6.5, no risk models: *α* = 36, *β* = 6.05. See Table D1 for details on how production and ecological efficiency were measured.

**Figure 4:**
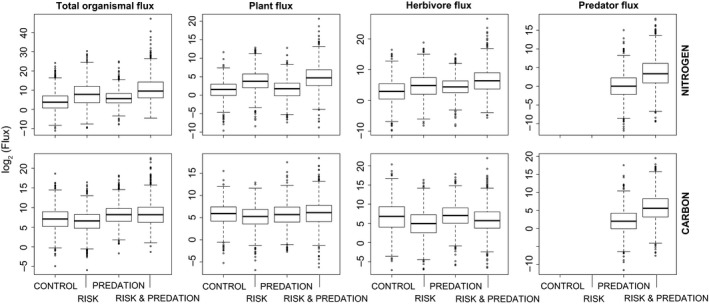
log_2_ of total, plant, herbivore, and predator nitrogen and carbon flux recycled within the ecosystem for four different models (“Control”* = *3‐level no risk, “Risk”* = *3‐level with risk, “Predation” = 4‐level no risk, “Risk & Predation” = 4‐level with risk). Results are for 1000 random parameter (uniform distribution) sets that meet feasibility conditions (i.e., equilibrium stocks of *N*_*i*_ and *C*_i_ > 0) and risk models: *α* = 36, *β* = 6.5, no risk models: *α* = 36, *β* = 6.05. See Table D1 for details on how flux was measured.

The “Risk” treatment led to higher soil N (1.08X) and plant N (4.5X) but lower herbivore N (0.65X) stocks than the “Control” whereas soil C (0.67X), plant C (0.62X), and herbivore C (0.7X) stocks were lower in the “Risk” treatment than the “Control” (Fig. 2). These differences, while small, may cause larger net differences to emerge at the ecosystem level (Hawlena et al. 2012), which is also evident in our other measures of ecosystem properties and functions (see below). Relative to the “Control”, the “Risk” treatment increased primary (70X) and secondary (1.95X) productivity and primary (4.7X) ecological efficiency, but lowered secondary (0.5X) ecological efficiency (Fig. 3) Compared to the “Control”, the “Risk” treatment had higher total N (16.7X) and lower total C (0.71X) recycled by organisms to the soil nutrient pools. Herbivores and plants accounted for most of the total N and C flux respectively (Fig. 4).

### Predation effects on ecosystem properties and functions

Relative to the “Control”, the “Predation” treatment caused an increase in soil C (2.43X), plant N (1.36X) and C (1.08X), and herbivore N (1.95X) and C (1.95X) and a small decrease in soil N (0.94X) (Fig. 2). The productivity and ecological efficiency patterns largely reflect the patterns in C and N stocks with “Predation” leading to higher primary (2.89X) and secondary (3.74X) production and primary (3.34X) and secondary (1.43X) ecological efficiency relative to the “Control” (Fig. 3). Total organismal N (3.66X) and C (2.15X) flux is higher in the “Predation” treatment compared to the “Control” and most of this flux is through the herbivore trophic level (Fig. 4).

### Comparing risk and predation effects on ecosystem properties and functions

The “Risk” treatment had higher soil N (1.15X) and plant N (3.31X) but lower soil C (0.27X), plant C (0.58X) and herbivore N (0.33X) and C (0.36X) relative to the “Predation” treatment (Fig. 2). Relative to the “Predation” treatment, the “Risk” treatment increased primary production (24X) and ecological efficiency (1.40X) but lowered secondary production (0.52X) and ecological efficiency (0.33X) (Fig. 3). Total N and C flux showed contrasting responses to “Risk” and “Predation” treatments with total N flux higher (4.57X) and total C flux lower (0.33X) in the “Risk” treatment compared to the “Predation” treatment (Fig. 4).

### Combined effects of risk and predation on ecosystem properties and functions

The combined “Risk & Predation” treatment led to synergistic effects that could not be predicted solely by summing the individual “Risk” and “Predation” effects. Specifically, “Risk & Predation” had much lower soil N (0.73X) and much higher plant N (8.89X) and soil C (2.19X) than the “Control”. In addition, predator N (16.11X) and C (17.31X) stocks were higher in the “Risk & Predation” treatment relative to the “Predation” only treatment (Fig. 2). Primary production (13.28X) and ecological efficiency (19.03X) and secondary production (13.35X) were much higher in “Risk & Predation” models than “Control”. The “Risk & Predation” model also led to higher tertiary production (35.28X) and ecological efficiency (2.96X) than the “Predation” only model (Fig. 3). Total N (55.39X) and C (2.13X) flux was higher in “Risk & Predation” model than the “Control”. Herbivores and plants accounted for most of the total N and C flux respectively (Fig. 4).

### Sensitivity of model results to changes in plant and herbivore C:N

Comparisons of ecosystem properties for our treatment contrasts (Risk vs. Control, Predation vs. Control, Risk & Predation vs. Control, and Risk & Predation vs. Predation) across all nine empirically‐based plant (*α*) and herbivore (*β*) C:N parameter sets (Table C1), showed little sensitivity. The outcomes were qualitatively similar to our mean plant and herbivore C:N parameter set (plant C:N* = *36, herbivore under risk C:N* = *6.5 and herbivores under no risk C:N* = *6.05) in 91% of all cases (see Figs. F1–F4). Standing stocks of C (82% qualitative concordance across parameter sets) and C recycled by organisms (85% qualitative concordance across parameter sets) were most sensitive to changes in the plant and herbivore C:N parameters (Figs. F1, F3). Production and respiration were least sensitive to changes in the plant and herbivore C:N parameters as all parameter sets were qualitatively concordant (Figs. F2, F4).

## Discussion

Our model explores the individual and combined effects of predator consumptive and nonconsumptive impacts on prey on ecosystem C and N cycling, relative to conditions where predators are absent. This exploration is motivated by empirical evidence that shows predator effects are manifest as changes in herbivore physiology, in addition to losses of herbivore biomass due to classic predator**–**prey consumptive interactions (Schmitz et al. 2010, 2014). Our model contributes to the growing body of theory (e.g., DeAngelis 1992; Loreau 1995; Loreau and Holt 2004; Gravel et al. 2010; Leroux et al.^2012^) which demonstrates that incorporating physical mass balance constraints in ecosystem trophic compartment models can lead to novel predictions at the ecosystem level. Our model differs, however, from previous advances that have explored consumer effects on elemental cycling (e.g., DeAngelis 1992; Loreau 1995, 2010; Loladze et al. 2000; Hall et al. 2007) in several important respects. First, we examine the fate of both C and N in response to top‐down feedbacks from predators, in addition to classic bottom‐up processes. Second, we explicitly account for the stocks and flows of both C and N throughout the ecosystem because predator effects mediated through prey physiology and stoichiometric means that C and N cycling can become uncorrelated (cf. Loladze et al. 2000; Hall et al. 2007). Finally, we examine how changes in prey C and N demand influences bottom‐up recycling feedbacks.

Infusing considerations of predation risk induced herbivore stress into ecosystem models requires allowing herbivores to have flexible physiological requirements for N and C that vary with the trophic structure of ecosystems (Hawlena and Schmitz 2010b; Leroux et al. 2012). This implies that C:N contents of herbivores are not fixed, for which there is emerging empirical support (Bertram et al. 2008; Persson et al. 2010). Also, C and N elements do not flow freely, but are bound up with other elements to form biochemicals such as proteins, lipids, and carbohydrates that comprise organic matter (Raubenheimer et al. 2009). In terrestrial plants, these soluble components are packaged within recalcitrant C‐based (e.g., cellulose, lignin, and fiber) structures used for plant support. Thus terrestrial plants may have high overall C content relative to N, but the fraction of total C that is soluble may be small (Robbins 1983; Karasov and Martinez del Rio 2007). So the quantity of soluble C that could be allocated to active metabolism can be highly limiting (Hall et al. 2007). These considerations of consumer and plant stoichiometry are the foundations on which we have built our models.

The analyses show that at steady state predators cause quantitative effects that differ from those found in model ecosystems without predators. Comparisons of models with predation and risk effects revealed that the predator risk effect was the fundamental and often quantitatively more important driver of shifts in C and N stocks, production rates and efficiencies, and recycling fluxes (Figs. 2, 3, 4). In some cases, risk and predation acted synergistically to influence ecosystem properties beyond their simple additive effects. For example, secondary production under the “Risk & Predation” treatment was 3.9X − 6.9X the secondary production under “Predation” and “Risk” treatments. In this case, predator consumptive and nonconsumptive effects combined to increase the quantity of nutrients flowing to higher trophic levels. Specifically, risk increases herbivore N recycling and predation removes herbivore N stocks therefore reducing herbivory. Our analysis suggests that in a material cycling modeling framework with physical mass balance constraints predator effects in ecosystems can be complex, involving interactions between consumptive and nonconsumptive effects on prey.

While consumptive and nonconsumptive predator effects are the driver of ecosystem properties and functions observed in our analysis, the host of resulting indirect feedbacks nonetheless emanate from interactions that happen at the plant‐herbivore interface. Specifically, physiological adjustments made by herbivores in response to perceived predation risk propagate downward in the food chain to affect plant and soil properties, as well as propagate upward to influence predator elemental balance. Thus, ecosystem properties and functions are neither top‐down nor bottom‐up controlled; but instead appear ultimately to be controlled from the middle‐out thereby blurring distinctions between top‐down and bottom‐up effects at the whole ecosystem level (Trussell and Schmitz 2012).

The nonconsumptive effects in our model were triggered by herbivore metabolic rate in response to perceived predation risk. Empirically, elevated herbivore metabolism enhances herbivore demand for plant soluble C and leads to the release of N (McPeek et al. 2001; Hawlena and Schmitz 2010b). The steady state conditions and sensitivity of our models reveal the outcome of this interaction. The greatest differences in elemental stocks and elemental fluxes among treatments occurred in the plant and herbivore trophic levels (Figs. 2, 4). Herbivores facing Risk predators had lower N stocks than herbivores facing Predation predators as well as no predators (Control), owing to release of N under risk. They also had lower C stocks than Predation and Control conditions owing to heightened C release via respiration. Plants, as a consequence show opposite trends in N. Increased herbivore demand for C is also reflected in lowest plant C stock under risk conditions. This effect is, however, offset by an interaction between predation and risk effects (cf. Predation vs. Risk & Predation treatments in Fig. 2). Overall, the effects of Risk had qualitatively opposite effects on elemental flux from all trophic compartments to the soil than Predation (Fig. 4).

While plant pools had much larger N contents with risk than without (Fig. 2), soil N tended to be invariant to treatment effects, implying that plants rapidly take up excess N released by stressed herbivores to the soil. The consequence of this “fast” nutrient cycling was both higher trophic transfer efficiency from the soil pool to plants and higher primary productivity in treatments with Risk effects than Control and Predation only conditions (Fig. 3).

Risk effects reduced trophic transfer efficiency to herbivores which translates to lower secondary producer efficiency. This emergent bottleneck in trophic transfer up the food chain, instigated from top‐down effects on herbivore physiology, is consistent with empirical findings and shows that predation risk may limit the length of food chains in ecosystems (sensu Trussell et al. 2006).

We are only now beginning to discover the nature of prey physiological plasticity in response to stress, and the stoichiometric mechanism we employ, while broadly applicable (Hawlena and Schmitz 2010b), is not universal. Some species compensate for risk by decreasing foraging effort and by altering food passage rate and assimilation, resulting in altered efficiency of N assimilation (Thaler et al. 2012; Dalton and Flecker 2014). Other species respond by enhancing N consumption and allocating it to build more musculature related to escape morphology (Costello and Michel 2013). Consideration of how these kinds of life‐history dependent plastic responses in prey body stoichiometry influence ecosystem properties and functioning would broaden the purview of how predator risk interacts with herbivore physiology to shape ecosystem functioning.

Nonetheless, consideration of plant‐herbivore stoichiometry in a food web context, especially, ironically *nontrophic* risk effects helps to appropriately account for the direct and indirect effects and feedbacks controlling elemental cycling within ecosystems (Leroux et al. 2012). Although we focus here on herbivore consumers, these principles generalize to intermediate consumers along detrital chains in ecosystems as well (Stief and Hölker 2006; Schmitz 2010; Baiser et al. 2011; Calizza et al. 2013; Zhao et al. 2013). Indeed, the biochemical machinery that permits such chronic stress responses is evolutionarily conservative and hence widespread across animal taxa (Hawlena and Schmitz 2010b; Boonstra 2013; Clinchy et al. 2013). Therefore, physiological plasticity in prey stoichiometry resulting from predation‐induced stress has the potential to provide general explanation for variation in C and N cycling.

## Conflict of Interest

None declared.

## Supporting information


**Appendix A.** Detailed conceptual diagram of our full ecosystem model.
**Appendix B.** Model analytical results.
**Appendix C.** Plant and herbivore C:N parameter sets used in our simulations.
**Appendix D**. Analytic formulas of ecosystem properties and functions.
**Appendix E.** Figure of herbivore respiration flux.
**Appendix F.** Sensitivity of model results to variation in plant and herbivore C:N.
