# Supplementary material for: Predator‐driven elemental cycling: the impact of predation and risk effects on ecosystem stoichiometry
Source: Ecol Evol. 2015 Oct 15;5(21):4976–88. doi: 10.1002/ece3.1760 (PMC4662303; doi:10.1002/ece3.1760)
Supplement: Supplementary file 1 — Appendix A. Detailed conceptual diagram of our full ecosystem model. Appendix B. Model analytical results. Appendix C. Plant and herbivore C:N parameter sets used in our simulations. Appendix D. Analytic formulas of ecosystem properties and functions. Appendix E. Figure of herbivore respiration flux. Appendix F. Sensitivity of model results to variation in plant and herbivore C:N. [file ECE3-5-4976-s001.docx]

**Online supplemental material:**

**Online Appendix A: Detailed conceptual diagram of our full ecosystem model**

**Online Appendix B: Model analytical results**

**Online Appendix C: Plant and herbivore C:N parameter sets used in our simulations**

**Online Appendix D: Analytic formulas of ecosystem properties and functions**

**Online Appendix E: Figure of herbivore respiration flux**

**Online Appendix F: Sensitivity of model results to variation in plant and herbivore C:N**

**Predator-driven elemental cycling: the impact of predation and risk effects on ecosystem stoichiometry**

Shawn J. Leroux^1^ and Oswald J. Schmitz^2^

^1^*Department of Biology, Memorial University of Newfoundland, St John’s, NL A1B 3X9 CANADA*

*^2^School of Forestry and Environmental Studies, Yale University,*

*New Haven, Connecticut, 06511 USA*

**Online Appendix A: Detailed conceptual diagram of our full ecosystem model**

**Figure A1** Conceptual diagram of general ecosystem model where resources flowing to herbivores are explicitly partitioned between active metabolism and growth and reproduction. We remove the consumptive effects of predators by removing the top trophic level. For models with predation risk *W_CH_ = 0* and for models without predation risk *W_NH_ = 0*. See Table 1 for variable and parameter definitions.

**Online Appendix B: Model analytical results**

**Equilibrium solutions of stoichiometrically-explicit ecosystem model**

Here, we provide the equilibrium solutions (where * denotes equilibrium stock) for our stoichiometrically-explicit ecosystem models. We present all equilibrium solutions for four model sets (i.e., treatments); 1) a herbivore-plant-soil model where predators heighten herbivore metabolism due to perceived predation risk (i.e., “Risk”); 2) a predator-herbivore-plant-soil model where predators cause direct biomass loss (i.e., “Predation”); 3) a predator-herbivore-plant-soil model with both predator-induced heightened herbivore metabolism and direct predation (i.e., “Risk & Predation”); and 4) a herbivore-plant-soil model without predator-induced heightened herbivore metabolism or direct predation (i.e., “Control”), i.e., a control for predator affects. Because we assume that plants, herbivores, and predators maintain C:N homeostasis (i.e., dC_P_/dt = *ψα*(dN_P_/dt); dC_H_/dt = *β*(dN_H_/dt); dC_D_/dt = *β*(dN_D_/dt)) our model can be reduced to five independent variables and their corresponding dynamical equations; dN_S_/dt (eq. 1a), dC_S_/dt (eq. 1b), dN_P_/dt (eq. 2a), dN_H_/dt (eq. 3a), dN_D_/dt (eq. 4a). We set the time derivatives of these five dynamical equations to zero and solve the system of equations for all equilibria. For our analyses, we focus on the single feasible equilibrium where all ecosystem compartments are positive (i.e., equilibrium C_i_ and N_i_ > 0). We present the feasibility conditions for this equilibrium point for each model.

**1) Solutions for “Risk” model:**

In a model without direct predation and with predation risk, W_CH_ = 0. There are 4 equilibrium points in the “Risk” model. Note that equilibrium 1-ii) = 3-ii), 1-iii) = 3-iii), and 1-iv) = 3-v) when N_D_ = C_D_ = 0.

*Equilibrium i)*

*Equilibrium ii)*

*Equilibrium iii)*

*Equilibrium iv)*

*Feasibility conditions of equilibrium 1) - iv)*

For 1 > *μ* > 0, 1 > τ > 0, 1 > *ψ* > 0, 1 > *ε* > 0, 1 > ρ > 0 and positive values for all other parameters, this equilibrium leads to positive stocks for all state variables if: and and . An additional constraint imposed by the conditions for herbivores to maintain homeostasis under predation risk by differential assimilation is that *β > ψα*.

**2) Solutions for “Predation” model:**

In a model with direct predation and without predation risk, W_NH_ = 0. There are 6 equilibrium points in the “Predation” model.

*Equilibrium i)*

*Equilibrium ii)*

*Equilibrium iii)*

*Equilibrium iv)*

*Equilibrium v)*

*Equilibrium vi)*

*Feasibility conditions of equilibrium 2) - vi)*

For 1 > *μ* > 0, 1 > *τ* > 0, 1 > *ψ* > 0, 1 > *ε* > 0, 1 > *ρ* > 0 and positive values for all other parameters, this equilibrium leads to positive stocks for all state variables if: . An additional constraint imposed by the conditions for herbivores to maintain homeostasis without predation risk by differential assimilation is that *β < ψα*.

**3) Solutions for “Risk & Predation” model:**

In a model with direct predation and predation risk, W_CH_ = 0. There are 6 equilibrium points in the “Risk & Predation” model.

*Equilibrium i)*

*Equilibrium ii)*

*Equilibrium iii)*

*Equilibrium iv)*

*Equilibrium v)*

*Equilibrium vi)*

*Feasibility conditions of equilibrium 3) - vi)*

For 1 > *μ* > 0, 1 > *τ* > 0, 1 > *ψ* > 0, 1 > *ε* > 0, 1 > *ρ* > 0 and positive values for all other parameters, this equilibrium leads to positive stocks for all state variables if: . An additional constraint imposed by the conditions for herbivores to maintain homeostasis under predation risk by differential assimilation is that *β > ψα*.

**4) Solutions for “Control” model:**

In a model without direct predation and without predation risk, W_NH_ = 0. There are 4 equilibrium points in the “Control” model. Note that equilibrium 2-ii) = 4-ii), 2-iii) = 4-iii), and 2-v) = 4-iv) when N_D_ = C_D_ = 0.

*Equilibrium i)*

*Equilibrium ii)*

*Equilibrium iii)*

*Equilibrium iv)*

*Feasibility conditions of equilibrium 4) - iv)*

For 1 > *μ* > 0, 1 > *τ* > 0, 1 > *ψ* > 0, 1 > *ε* > 0, 1 > *ρ* > 0 and positive values for all other parameters, this equilibrium leads to positive stocks for all state variables if: . An additional constraint imposed by the conditions for herbivores to maintain homeostasis without predation risk by differential assimilation is that *β < ψα*.

**Online Appendix C: Plant and herbivore C:N parameter sets used in our simulations**

**Table C1** Empirically-derived plant (α) and herbivore (β) C:N parameter sets used in our simulations. Elser et al. (2000) reported a mean terrestrial plant C:N = 36 (s.d. = 23) and a mean terrestrial invertebrate herbivore C:N = 6.5 (s.d. = 1.9). Syntheses of C:N for risk vs no risk conditions are unavailable, but Hawlena and Schmitz (2010b) reported mean terrestrial invertebrate (i.e., grasshopper) C:N under no risk that is 0.93 x than with risk (C:N of 4.0 vs 4.3). We set plant C:N to Elser et al. (2000) mean terrestrial plant C:N (i.e., 36) + 1 s.d. (i.e., 59) and – 1 s.d. (i.e., 13). We set herbivore C:N under risk to Elser et al. (2000) mean terrestrial invertebrate herbivore C:N (i.e., 6.5) + 1 s.d. (i.e., 8.4) and – 1 s.d. (i.e., 4.6) and herbivore C:N without risk to 0.93X herbivore C:N under risk (i.e., 6.05, 4.275, and 7.8). In-text results are reported for the “mean” parameter set 1. Results for other parameters sets are summarized in Appendix F.

| **Parameter set** | **Models with Risk (i.e., “Risk” and “Risk & Predation”)** | | **Models without Risk (i.e., “Control” and “Predation”** | |
| --- | --- | --- | --- | --- |
|  | **Plant** (α) | **Herbivore** (β) | **Plant** (α) | **Herbivore** (β) |
| **1 ("mean”)** | 36 | 6.5 | 36 | 6.05 |
| **2** | 36 | 4.6 | 36 | 4.275 |
| **3** | 36 | 8.4 | 36 | 7.8 |
| **4** | 13 | 6.5 | 13 | 6.05 |
| **5** | 13 | 4.6 | 13 | 4.275 |
| **6** | 13 | 8.4 | 13 | 7.8 |
| **7** | 59 | 6.5 | 59 | 6.05 |
| **8** | 59 | 4.6 | 59 | 4.275 |
| **9** | 59 | 8.4 | 59 | 7.8 |

**Online Appendix D: Analytic formulas of ecosystem properties and functions**

**Table D1** Formulas used to calculate total flux of nitrogen and carbon recycled to the soil nutrient pools within the ecosystem, and trophic-level specific contributions as well as production and ecological efficiency of each trophic level. N_i_ and C_i_ are equilibrium stocks.

| **Trophic level** | **N Recycling Flux** | | **C Recycling Flux** | | **Production** | **Ecological efficiency** |
| --- | --- | --- | --- | --- | --- | --- |
|  | **Risk** | **No Risk** | **Risk** | **No Risk** |
| Whole ecosystem | *r_P_N_P_ + ρa_H_N_P_N_H_ + r_H_N_H_ + (1 - ρ) a_H_N_P_N_H_ ((β -ψα)/β) + r_D_N_D_ + εa_D_N_H_N_D_* | *r_P_N_P_ + ρa_H_N_P_N_H_ + r_H_N_H_ + r_D_N_D_ + εa_D_N_H_N_D_* | *ψαr_P_N_P_ + βr_H_N_H_ + (1 - μ) ρψαa_H_N_P_N_H_ + βr_D_N_D_ + (1 - τ) εβa_D_N_H_N_D_* | *ψαr_P_N_P_ + βr_H_N_H_ + (1 - μ) ρψαa_H_N_P_N_H_ + βr_D_N_D_ + (1 - τ) εβa_D_N_H_N_D_* |  |  |
| Plants | *r_P_N_P_* | *r_P_N_P_* | *ψαr_P_N_P_* | *ψαr_P_N_P_* | *a_P_C_S_N_S_N_P_* | *a_P_C_S_N_S_N_P_/I* |
| Herbivores  (Secondary) | *ρa_H_N_P_N_H_ + r_H_N_H_ + (1 - ρ) a_H_N_P_N_H_ ((β -ψα)/β)* | *ρa_H_N_P_N_H_ + r_H_N_H_* | *βr_H_N_H_ + (1 - μ) ρψαa_H_N_P_N_H_* | *βr_H_N_H_ + (1 - μ) ρψαa_H_N_P_N_H_* | *(1 - ρ) a_H_N_P_N_H_* | *(1 - ρ) a_H_N_P_N_H_/ a_P_C_S_N_S_N_P_* |
| Predators  (Tertiary) | *r_D_N_D_ + εa_D_N_H_N_D_* | *r_D_N_D_ + εa_D_N_H_N_D_* | *βr_D_N_D_ + (1 - τ)εβa_D_N_H_N_D_* | *βr_D_N_D_ + (1 - τ)εβa_D_N_H_N_D_* | *(1 - ε)a_D_N_H_N_D_* | *(1 - ε) a_D_N_H_N_D_ /(1 - ρ) a_H_N_P_N_H_* |

**Online Appendix E: Figure of herbivore respiration flux**

**Figure E1** Log_2_ of herbivore respiration flux for four different models (“Control” = 3-level no risk, “Risk” = 3-level with risk, “Predation” = 4-level no risk, “Risk & Predation” = 4-level with risk). Results are for 1,000 random parameter (uniform distribution) sets that meet feasibility conditions (i.e., equilibrium stocks of N_i_ and C_i_ > 0) and risk models: α = 36, β = 6.5, no risk models: α = 36, β = 6.05.

**Online Appendix F: Sensitivity of model results to variation in plant and herbivore C:N**

Here we show results for all 9 plant and herbivore C:N parameter sets (see Table C1 for specific C:N parameter sets used and main text for a description of these parameters). These figure depict how sensitive our results are to variations in plant and herbivore C:N. We provide 4 treatment contrasts: i) Risk vs Control (Risk/Control), ii) Predation vs Control (Predation/Control), iii) Risk & Predation vs Control (Risk & Predation/Control, and iv) Risk & Predation vs Predation (Risk & Predation/Predation). Magnitudes greater than 1 indicate a positive effect of a treatment on an ecosystem property relative to another treatment and magnitudes less than 1 indicate a negative effect. This qualitative breakpoint is depicted by a solid vertical line in all figures.

**Figure F1** Ratio of median log_2_ mass of soil, plant, herbivore and predator N (top panels) and C (bottom panels) in Risk vs Control, Predation vs Control, Risk & Predation vs Control, and Risk & Predation vs Predation treatments. Each circle represents 1 of 9 plant and herbivore C:N parameter combinations (see Table C1). Results are for 1,000 random parameter (uniform distribution) sets that meet feasibility conditions (i.e., equilibrium stocks of N_i_ and C_i_ > 0).

**Figure F2** Ratio of median log_10_ primary, secondary and tertiary production (top panels) and efficiency (bottom panels) in Risk vs Control, Predation vs Control, Risk & Predation vs Control, and Risk & Predation vs Predation treatments. Each circle represents 1 of 9 plant and herbivore C:N parameter combinations (see Table C1). Results are for 1,000 random parameter (uniform distribution) sets that meet feasibility conditions (i.e., equilibrium stocks of N_i_ and C_i_ > 0).

**Figure F3** Ratio of median log_2_ plant, herbivore, predator and total N flux (top panels) and C flux (bottom panels) in Risk vs Control, Predation vs Control, Risk & Predation vs Control, and Risk & Predation vs Predation treatments. Each circle represents 1 of 9 plant and herbivore C:N parameter combinations (see Table C1). Results are for 1,000 random parameter (uniform distribution) sets that meet feasibility conditions (i.e., equilibrium stocks of N_i_ and C_i_ > 0).

**Figure F4** Ratio of median log_2_ herbivore respiration in Risk vs Control, Predation vs Control, Risk & Predation vs Control, and Risk & Predation vs Predation treatments. Each circle represents 1 of 9 plant and herbivore C:N parameter combinations (see Table C1). Results are for 1,000 random parameter (uniform distribution) sets that meet feasibility conditions (i.e., equilibrium stocks of N_i_ and C_i_ > 0).
